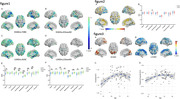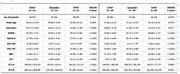# Synapse loss in early‐ and late‐onset Alzheimer's Disease assessed by ^18^F‐SynVest‐1

**DOI:** 10.1002/alz.085609

**Published:** 2025-01-09

**Authors:** kun he, Fang Xie, Qi Huang

**Affiliations:** ^1^ Huashan hospital affiliated to fudan university, Shanghai, shanghai China; ^2^ Huashan Hospital, Fudan University, Shanghai, Shanghai China; ^3^ Huashan Hospital, Shanghai, shanghai China

## Abstract

**Background:**

We aimed to investigate the loss of synaptic density in early‐onset and late‐onset Alzheimer's Disease.

**Method:**

One hundred and eighty‐two participants underwent synaptic density PET with ^18^F‐SynVesT‐1. Including 23 early‐onset Alzheimer‘s Disease (EOAD), 58 late‐onset Alzheimer's Disease (LOAD), 16 EOnonAD, 28 LOnonAD, 31 younger normal control (age < 65) and 26 older normal control (age ⩾ 65). We analyzed the synaptic density loss in EOAD and LOAD relative to their control group respectively. Associations between age and synaptic loss were evaluated in different groups.

**Result:**

EOAD and LOAD groups both displayed significant synaptic density across most areas of cerebrum cortex. Relative to LOAD group, EOAD group showed severer synaptic loss in Lateral Parietal lobe. In EOAD group, synaptic density in occipital lobe had positive association with age. While, in LOAD group, synaptic density in most areas of the cerebrum cortex had a negative association with age.

**Conclusion:**

Our study suggested that the areas of synaptic loss were different between EOAD and LOAD.